# Trends of Dispensed Opioids in Catalonia, Spain, 2007–19: A Population-Based Cohort Study of Over 5 Million Individuals

**DOI:** 10.3389/fphar.2022.912361

**Published:** 2022-06-08

**Authors:** Junqing Xie, Victoria Y. Strauss, Gary S. Collins, Sara Khalid, Antonella Delmestri, Aleksandra Turkiewicz, Martin Englund, Mina Tadrous, Carlen Reyes, Daniel Prieto-Alhambra

**Affiliations:** ^1^ Pharmaco- and Device Epidemiology, Centre for Statistics in Medicine, Nuffield Department of Orthopaedics, Rheumatology and Musculoskeletal Sciences, Botnar Research Centre, University of Oxford, Oxford, United Kingdom; ^2^ Centre for Statistics in Medicine, Nuffield Department of Orthopaedics, Rheumatology & Musculoskeletal Sciences, University of Oxford, Oxford, United Kingdom; ^3^ NIHR Oxford Biomedical Research Centre, John Radcliffe Hospital, Oxford, United Kingdom; ^4^ Clinical Epidemiology Unit, Orthopaedics, Clinical Sciences, Lund, Lund University, Lund, Sweden; ^5^ Leslie Dan Faculty of Pharmacy, University of Toronto, Toronto, ON, Canada; ^6^ Centro de Investigación Biomédica en Red Fragilidad y Envejecimiento Saludable (CIBERFes), Instituto Carlos III, Madrid, Spain; ^7^ Fundació Institut Universitari per a La Recerca a L’Atenció Primària de Salut Jordi Gol I Gurina (IDIAPJGol), Barcelona, Spain

**Keywords:** analgesic opioid, drug utilization, observational study, primary health care, Spain

## Abstract

**Objective:** To characterize the trend of opioid use (number of users, dispensations and oral morphine milligram equivalents) in Catalonia (Spain).

**Design, setting, and participants:** This population-based cohort study included all individuals aged 18 years or older, registered in the Information System for Research in Primary Care (SIDIAP), which covers >75% of the population in Catalonia, Spain, from 1 January 2007, to 31 December 2019.

**Main exposure and outcomes:** The exposures were all commercialized opioids and their combinations (ATC-codes): codeine, tramadol, oxycodone, tapentadol, fentanyl, morphine, and other opioids (dihydrocodeine, hydromorphone, dextropropoxyphene, buprenorphine, pethidine, pentazocine). The main outcomes were the annual figures per 1,000 individuals of 1) opioid users, 2) dispensations, and 3) oral morphine milligram equivalents (MME). Results were stratified separately by opioid types, age (5-year age groups), sex (male or female), living area (rural or urban), and socioeconomic status (from least, U1, to most deprived, U5). The overall trends were quantified using the percentage change (PC) between 2007 and 2019.

**Results:** Among 4,656,197 and 4,798,114 residents from 2007 to 2019, the number of opioid users, dispensations and morphine milligram equivalents per 1,000 individuals increased 12% (percentage change: 95% confidence interval (CI) 11.9–12.3%), 105% (95% confidence interval 83%–126%) and 339% (95% CI 289%–390%) respectively. Tramadol represented the majority of opioid use in 2019 (61, 59, and 54% of opioid users, dispensations, and total MME, respectively). Individuals aged 80 years or over reported the sharpest increase regarding opioid users (PC: 162%), dispensations (PC: 424%), and MME (PC: 830%). Strong opioids were increasingly prescribed for non-cancer pains over the years.

**Conclusion:** Despite the modest increase of opioid users, opioid dispensations and MME increased substantially, particularly in the older population. In addition, strong opioids were incrementally indicated for non-cancer pains over the years. These findings suggest a transition of opioid prescriptions from intermittent to chronic and weak to strong and call for more rigorous opioid stewardship.

## Introduction

There has been an increasing awareness of the risks associated with opioid use; the 2019 World Drug Report (UN Office on Drugs and Crime, 2019) stated that 35 million people worldwide suffered from drug disorders, and two-thirds of the deaths related to drug disorders were caused by opioids (UN Office on Drugs and Crime, 2019). The United States (US) is the single country which seems to be the most affected, with 4% of its population using opioids (UN Office on Drugs and Crime, 2019) and with an increase in its age-adjusted rate of drug overdose deaths, from 0.3 to 9.9 per 100,000 standard population between 1999 and 2018 (Hedegaard et al., 2020). In Europe, opioid consumption figures are lower than in the US. However, the rapid increase of opioid prescriptions (i.e. 34% opioid prescription increase in England between 1998 and 2016 and a two-fold increase of opioid users in the Netherlands between 2008 and 2017) (Curtis et al., 2019; Kalkman et al., 2019) foreshadows a future public health problem.

Previous European reports assessing opioid consumption are highly heterogeneous in their methods and on the opioids analyzed (Hider-Mlynarz et al., 2018; Bosetti et al., 2019), making comparison difficult. Although two previous studies reported an increase in opioid use in the past years in Spain (Herrera-Gómez et al., 2019; Hurtado et al., 2020), they were subject to several limitations, such as no data on critical sub-populations and opioid doses measured by morphine milligram equivalents. Moreover, none of them analyzed the opioid indications and prescribing patterns (Domínguez-Berjón et al., 2008; Hurtado et al., 2020). Therefore, this study aims to comprehensively characterize the trend of opioid use (number of users, dispensations and MME) in Catalonia (Spain).

## Methods

### Data Sources and Study Design

A population-based cohort study was conducted using data from the Information System for Research in Primary Care (SIDIAP), a research database including demographic information (age, sex), socioeconomic status through the MEDEA deprivation index (Mortalidad en áreas pequeñas Españolas y Desigualdades Socioeconómicas y Ambientales) (Domínguez-Berjón et al., 2008), as well as routinely collected primary care data such as diagnosis (ICD-10 codes), referrals, laboratory tests, prescriptions and drug dispensations through community pharmacy linkage. The Spanish national health care system guarantees universal health coverage to all Spanish residents. Patients are allocated to a primary health care center and to health care professionals (medicine and nursery) depending on their place of residency. The SIDIAP database covers 279 primary care practices in Catalonia and about 6 million residents (∼75% of the Catalan population). Both drug prescriptions and dispensations are coded with the Anatomical Therapeutic Chemical (ATC) system (https://www.whocc.no/). The validity of SIDIAP has been previously established (Ramos et al., 2012; Ponjoan et al., 2019), and the SIDIAP data have been used to conduct multiple drug utilization studies, including regulatory requests (Berencsi et al., 2020).

We included all participants in the SIDIAP database aged 18 years or older from 1 January 2007, to 31 December 2019, and used individual-level data for all analyses.

### Opioids Classification

We quantified opioids use based on pharmacies dispensations rather than on general practitioners (GP) prescriptions to better reflect the actual opioid consumption. We identified all types of opioids and their combinations by 7-digit ATC codes. We grouped opioids by ingredient: codeine [R05DA04, N02AA59, N02AJ06, N02AJ07, N02AJ08], tramadol [N02AX02, N02AJ13, N02AJ14], oxycodone [N02AA05, N02AA55], tapentadol [N02AX06], fentanyl [N02AB03], and morphine [N02AA01]. All additional opioids were combined in the category of *other opioids*, including dihydrocodeine [N02AA08], hydromorphone [N02AA03], dextropropoxyphene [N02AC04], buprenorphine [N02AE01], pethidine [N02AB02], and pentazocine [N02AD01].

For each dispensation of opioids, we extracted the total dose, unit, and dispensation date and converted the original opioids dose to oral morphine milligram equivalents (MME) based on prespecified conversion factors (see appendix).

### Statistical Analyses

We calculated the following summary measures to assess yearly opioid use (over 12 months from 1st January to 31st December): 1) number of opioid users per 1,000 individuals, 2) number of opioid dispensations per 1,000 individuals, and 3) oral MME per 1,000 individuals. Accordingly, the numerators were calculated as the number of unique individuals who had received at least one opioid dispensation, number of any opioid dispensations, and total oral MME of any opioid dispensations during the index year. The denominator is the number of eligible individuals (defined as those registered in the database up to the first day of the index year) and is the same for the three quantities. We calculated each measure in the overall population (total opioids and each opioid subtype) and subgroups stratified by age (5-year age groups from 18 up to 80), sex (male or female), living area (rural or urban), and socioeconomic status (MEDEA) which was divided into fifths: the first representing the least deprived (U1), and the last (U5) the most deprived. To adjust for demographic changes over time, an age direct standardization was performed using the 2007 population as the reference with 5-years age bands. We quantified the secular trend between 2007 and 2019 with percentage change (PC) using the formula (value in 2019 - value in 2007)/(value in 2007) * 100 and calculated its 95% confidence intervals based on Poisson regressions with robust standard errors (Patil and Kulkarni, 2012).

We estimated the relative proportions of nine pain-related conditions (cancer, back pain, neck pain, osteoarthritis, fibromyalgia, cough, fracture, falls, surgery) as potential clinical indications occurring in the year before the opioid dispensation. Specifically, in each year, we defined the first opioid dispensation as the index date and categorized people as incident or prevalent users depending on whether they were dispensed the same opioid 1 year before the index date. Users can have multiple indications if relevant diagnoses were identified on or 1-year before the index date, but each indication was only counted once for each person. These conditions were prespecified a priori through operational definitions using ICD-10 codes (Supplementary Appendix Table SA1). All analyses were conducted with R4.04.

### Patient and Public Involvement

This study used routinely collected health data. Although study participants contributed in important ways to this research, no patients were involved in the design, conduct, reporting, or dissemination plans of our research. Some of the co-authors are healthcare workers and therefore represented in some of our analyses.

## Results

### Overall Trends

The size and composition of the population covered by SIDIAP remained stable between 2007 and 2019 (Supplementary Appendix Table SA2). There were 4,656,197 participants in 2007 vs. 4,798,114 in 2019. The mean age increased from 47.3 (SD: 18.4) in 2007 to 50.4 (SD:18.6) in 2019. The proportion of males and females were similar over the study period, with 510 females per 1,000 people and 512 females per 1,000 people in 2007 and 2019, respectively.

**FIGURE 1:**
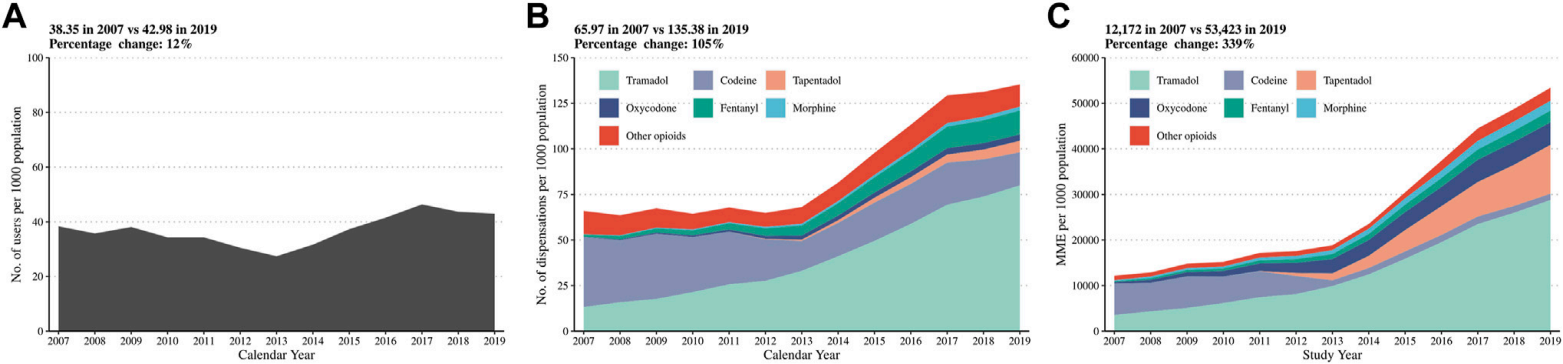
Number of opioid users **(A)**, number of opioid dispensations **(B)**, and oral MME **(C)** per 1,000 population from 2007 to 2019 in the overall population. MME: Morphine milligram equivalents. Other opioids included dihydrocodeine [N02AA08], hydromorphone [N02AA03], dextropropoxyphene [N02AC04], buprenorphine [N02AE01], pethidine [N02AB02], and pentazocine [N02AD01].

In Figure 1 and Table 1, the number of people with opioid dispensations increased by 12% (from 38.4 per 1,000 in 2007 to 43.0 per 1,000 individuals in 2019, *p* < 0.01) during the study period. After counting for multiple dispensations per person during the year and correcting for opioid strength, there was a 105% increase in the number of opioid dispensations (from 66.0 per 1,000 to 135.4 per 1,000 individuals, *p* < 0.01) and a 339% increase in MME (from 12,172 to 53,423 mg per 1,000 individuals, *p* < 0.01). After age-standardization, the increasing trend of opioid use was mitigated to 3, 82, and 282% accordingly. Of note, the opioid dispensations and MME per opioid user increased from 1.7 to 3.1 and 309 to 1,350 respectively between 2007 and 2019 (Supplementary Appendix Figure A1).

**TABLE 1:** Use of opioids and their subtypes, measured by different metrics from 2007 to 2019.

Study year	Opioids	Opioids (AS)	Tramadol	Codeine	Tapentadol	Oxycodone	Fentanyl	Morphine	Others
Number of Users per 1,000 Population									
2007	38.35	38.35	7.47 (0.19)	31.52 (0.82)	NA	0.04 (0)	0.41 (0.01)	0.09 (0)	11 (0.29)
2008	35.74	35.74	8.36 (0.23)	27.83 (0.78)	NA	0.12 (0)	0.55 (0.02)	0.15 (0)	9.38 (0.26)
2009	38.09	38.06	9.06 (0.24)	29.47 (0.77)	NA	0.15 (0)	0.64 (0.02)	0.19 (0)	9.07 (0.24)
2010	34.31	34.16	10.12 (0.29)	24.57 (0.72)	NA	0.17 (0)	0.69 (0.02)	0.21 (0.01)	7.1 (0.21)
2011	34.31	33.98	11.06 (0.32)	23.5 (0.68)	0.02 (0)	0.23 (0.01)	0.82 (0.02)	0.24 (0.01)	6.57 (0.19)
2012	30.49	29.86	11.55 (0.38)	18.85 (0.62)	0.09 (0)	0.32 (0.01)	0.93 (0.03)	0.3 (0.01)	6.3 (0.21)
2013	27.40	26.53	13.2 (0.48)	13.62 (0.5)	0.21 (0.01)	0.41 (0.01)	1.14 (0.04)	0.34 (0.01)	7.8 (0.28)
2014	31.66	30.42	15.78 (0.5)	15.14 (0.48)	0.42 (0.01)	0.47 (0.01)	1.38 (0.04)	0.41 (0.01)	8.73 (0.28)
2015	37.31	35.53	18.66 (0.5)	17.67 (0.47)	0.74 (0.02)	0.52 (0.01)	1.67 (0.04)	0.49 (0.01)	10.57 (0.28)
2016	41.51	39.29	21.71 (0.52)	18.52 (0.45)	0.93 (0.02)	0.59 (0.01)	1.95 (0.05)	0.56 (0.01)	12.05 (0.29)
2017	46.37	43.44	25.42 (0.55)	19.56 (0.42)	1.08 (0.02)	0.64 (0.01)	2.19 (0.05)	0.64 (0.01)	13.03 (0.28)
2018	43.67	40.36	25.04 (0.57)	17.06 (0.39)	1.26 (0.03)	0.6 (0.01)	2.23 (0.05)	0.62 (0.01)	11.45 (0.26)
2019	42.98	39.33	26.23 (0.61)	14.96 (0.35)	1.41 (0.03)	0.57 (0.01)	2.28 (0.05)	0.62 (0.01)	10.13 (0.24)
Percentage change	12	3	251	-53	6,950	1,325	456	589	-8
**Number of dispensations per 1,000 population**									
2007	65.97	65.97	13.22 (0.2)	38.41 (0.58)	NA	0.18 (0)	1.21 (0.02)	0.18 (0)	12.77 (0.19)
2008	63.57	63.58	15.84 (0.25)	34.05 (0.54)	NA	0.48 (0.01)	1.84 (0.03)	0.36 (0.01)	11 (0.17)
2009	67.42	67.31	17.67 (0.26)	35.75 (0.53)	NA	0.69 (0.01)	2.26 (0.03)	0.44 (0.01)	10.63 (0.16)
2010	64.43	63.91	21.43 (0.33)	30.22 (0.47)	NA	0.93 (0.01)	2.73 (0.04)	0.49 (0.01)	8.64 (0.13)
2011	67.86	66.64	25.67 (0.38)	28.86 (0.43)	0.03 (0)	1.18 (0.02)	3.44 (0.05)	0.59 (0.01)	8.07 (0.12)
2012	64.89	62.81	27.59 (0.43)	22.75 (0.35)	0.29 (0)	1.6 (0.02)	4.22 (0.07)	0.76 (0.01)	7.69 (0.12)
2013	68.1	64.88	33.07 (0.49)	16.41 (0.24)	0.73 (0.01)	2.24 (0.03)	5.52 (0.08)	0.94 (0.01)	9.2 (0.14)
2014	81.51	76.75	41.06 (0.5)	18.31 (0.22)	1.46 (0.02)	2.53 (0.03)	6.82 (0.08)	1.16 (0.01)	10.15 (0.12)
2015	97.81	91.01	49.48 (0.51)	21.04 (0.22)	2.66 (0.03)	2.81 (0.03)	8.37 (0.09)	1.34 (0.01)	12.11 (0.12)
2016	113.22	104.31	58.82 (0.52)	21.98 (0.19)	3.66 (0.03)	3.11 (0.03)	10.15 (0.09)	1.56 (0.01)	13.95 (0.12)
2017	129.34	117.57	69.34 (0.54)	23.16 (0.18)	4.39 (0.03)	3.48 (0.03)	11.85 (0.09)	1.9 (0.01)	15.24 (0.12)
2018	131.27	117.68	73.81 (0.56)	20.44 (0.16)	5.35 (0.04)	3.53 (0.03)	12.55 (0.1)	2.03 (0.02)	13.57 (0.1)
2019	135.38	120.26	79.95 (0.59)	18.2 (0.13)	6.28 (0.05)	3.5 (0.03)	13.13 (0.1)	2.1 (0.02)	12.22 (0.09)
Percentage change	105	82	505	-53	20,833	1844	985	1,067	-4
**Oral morphine milligram equivalents per 1,000 population**									
2007	12,172	12,172	3527 (0.29)	6,937 (0.57)	NA	290 (0.02)	220 (0.02)	191 (0.02)	1,006 (0.08)
2008	12,888	12,886	4,357 (0.34)	6,264 (0.49)	NA	646 (0.05)	363 (0.03)	370 (0.03)	889 (0.07)
2009	14,814	14,755	5,081 (0.34)	6,944 (0.47)	NA	922 (0.06)	479 (0.03)	395 (0.03)	993 (0.07)
2010	15,211	15,026	6,166 (0.41)	5,836 (0.38)	NA	1,221 (0.08)	542 (0.04)	421 (0.03)	1,026 (0.07)
2011	17,178	16,766	7,447 (0.43)	5,684 (0.33)	64 (0)	1,677 (0.1)	676 (0.04)	601 (0.03)	1,029 (0.06)
2012	17,576	16,866	8125 (0.46)	3991 (0.23)	651 (0.04)	2,227 (0.13)	823 (0.05)	691 (0.04)	1,070 (0.06)
2013	18,852	17,677	9,891 (0.52)	1,280 (0.07)	1,505 (0.08)	3150 (0.17)	1,083 (0.06)	886 (0.05)	1,056 (0.06)
2014	23,526	21,715	12,446 (0.53)	1,421 (0.06)	2,657 (0.11)	3476 (0.15)	1,326 (0.06)	1,158 (0.05)	1,044 (0.04)
2015	30,461	27,757	15,880 (0.52)	1,622 (0.05)	4,673 (0.15)	3946 (0.13)	1,624 (0.05)	1,382 (0.05)	1,333 (0.04)
2016	37,404	33,572	19,505 (0.52)	1,603 (0.04)	6,316 (0.17)	4,219 (0.11)	1961 (0.05)	1,588 (0.04)	2,213 (0.06)
2017	44,488	39,391	23,481 (0.53)	1,681 (0.04)	7,557 (0.17)	4,870 (0.11)	2,268 (0.05)	1890 (0.04)	2,741 (0.06)
2018	48,773	42,747	25,970 (0.53)	1,510 (0.03)	9,028 (0.19)	5,023 (0.1)	2,448 (0.05)	2035 (0.04)	2,758 (0.06)
2019	53,423	46,486	28,776 (0.54)	1,390 (0.03)	10,712 (0.2)	4,926 (0.09)	2,602 (0.05)	2,149 (0.04)	2,870 (0.05)
Percentage change	339	282	716	-80	16,638	1,599	1,083	1,025	185

AS: Age-standardised. The numbers in the bracket are the proportions of each opioid type. The sum of proportions in each year for the opioid user metrics may be greater than 1. The percentage change for tapentadol was calculated between 2011 and 2019. Other opioids included dihydrocodeine [N02AA08], hydromorphone [N02AA03], dextropropoxyphene [N02AC04], buprenorphine [N02AE01], pethidine [N02AB02], and pentazocine [N02AD01]. NA: not available for the opioid type in Catalonia.

The number of opioid users per 1,000 individuals and the number of opioid dispensations per 1,000 individuals remained constant until 2013, after which, it increased sharply until the end of the study period. The increase from 2013 was driven mainly by the use of tramadol (Figure 1). In 2007, codeine was the most dispensed opioid (31.5 users and 38.4 dispensations per 1,000 individuals), followed by tramadol (7.5 users and 13.2 dispensations per 1,000 population). In comparison, in 2019, tramadol users increased to 26.2 per 1,000 individuals and dispensations to 80.0 per 1,000 individuals, making up 61 and 59% of all opioid users and dispensations (Table 1).

When considering oral MME, opioid use seemed to continuously increase, primarily due to a rise in tramadol use from MME 3,527 per 1,000 individuals in 2007 to MME 28,776 per 1,000 individuals in 2019, making up 50% of total oral MME (Table 1).

### Subgroups

**FIGURE 2:**
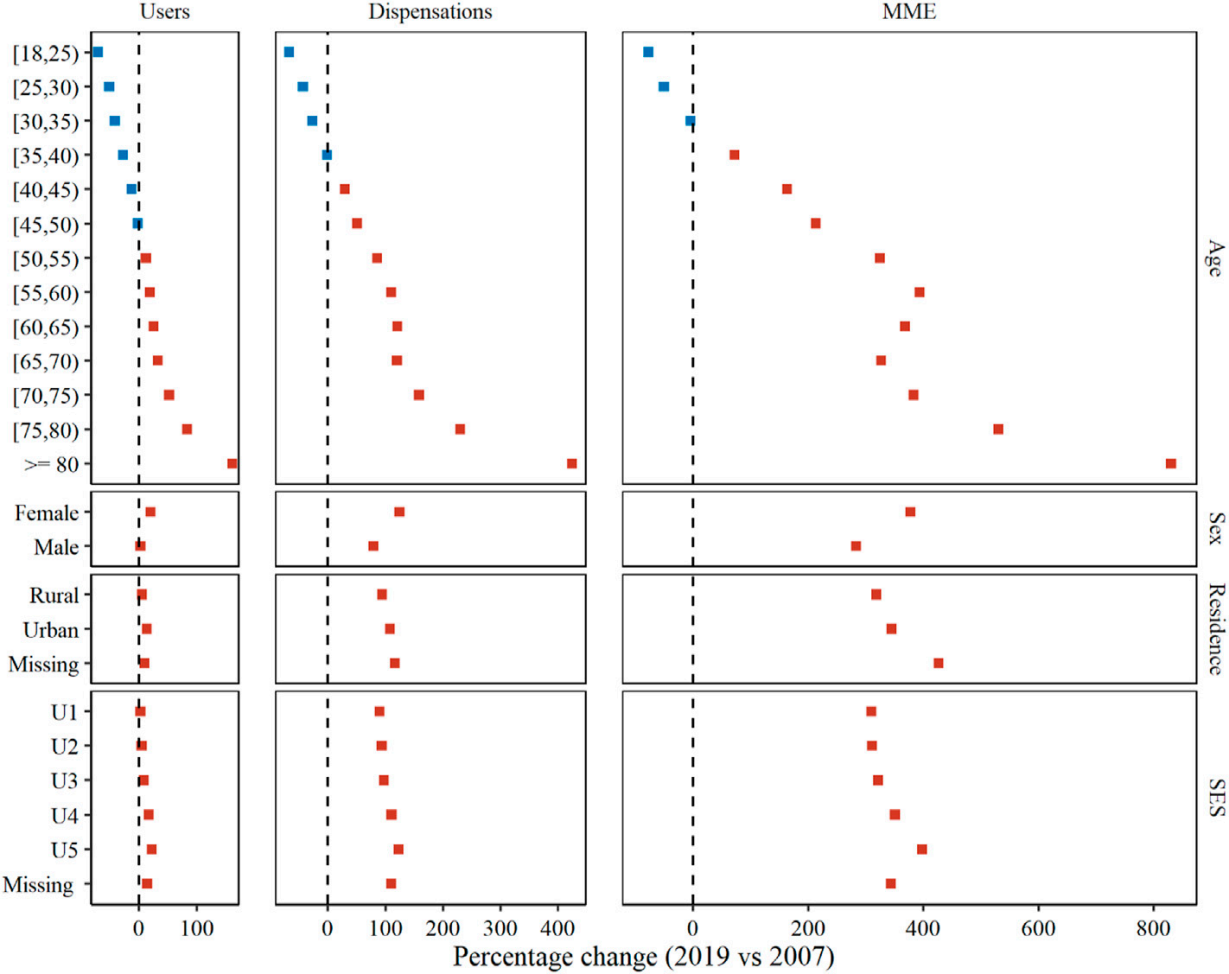
Percentage changes of opioids use between 2007 and 2019 across subgroups.

Between 2007 and 2019, there was an age-dependent shift in opioid use, with a slight decrease among younger adults but a dramatic increase among the elderly. For example, opioid users aged 18–24 years old decreased from 36.4 in 2007 to 10.5 in 2019 per 1,000 individuals (PC -71.1%); but increased from 27.5 in 2007 to 72.0 in 2019 per 1,000 individuals (PC 162.2%) in ≥80-year-old (Figure 2, Supplementary Appendix Table SA3). Also, 2019 than 2007 had consistently higher rates of opioid users for other subgroups, with PC of 19.6% 13.9 and 21.8% in female, urban residents and the most deprived subgroup respectively. More pronounced trends were observed across all studied subgroups in terms of opioid dispensations and MME.

### Pain-Related Diagnoses Among Opioid Users

**FIGURE 3:**
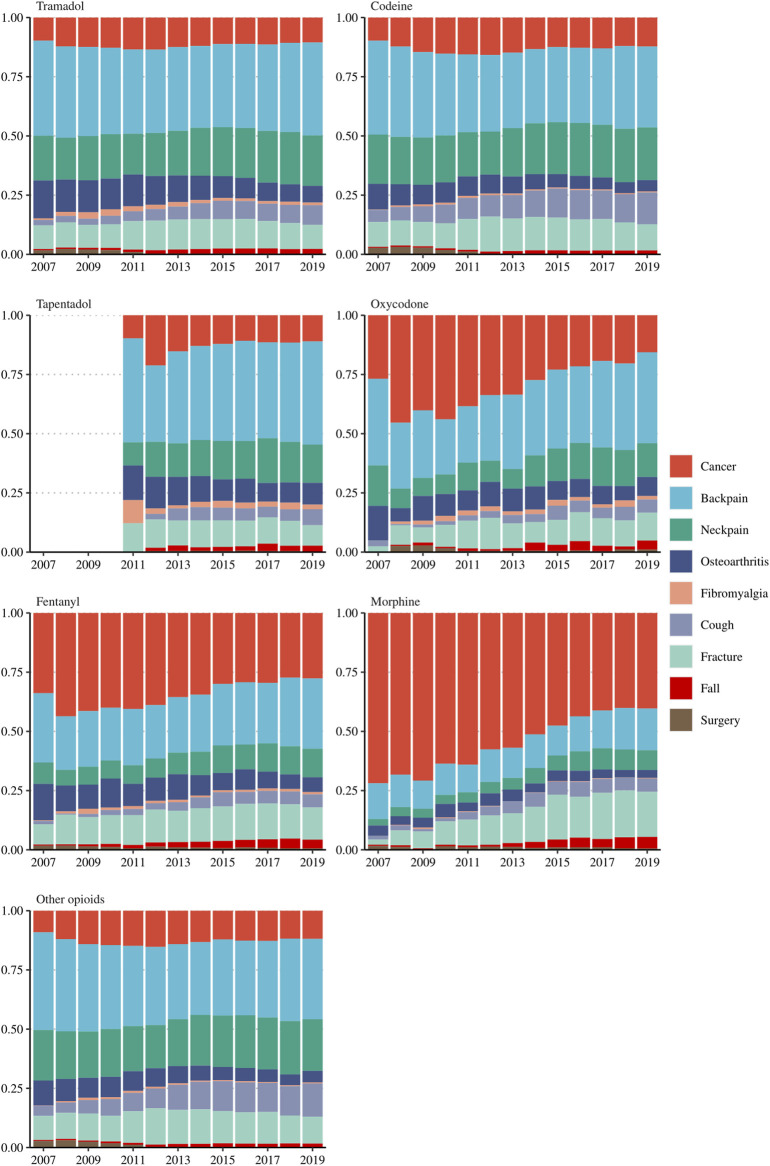
The proportion of nine pain-related diagnoses among incident opioid users, stratified by opioid subtypes. Other opioids included dihydrocodeine [N02AA08], hydromorphone [N02AA03], dextropropoxyphene [N02AC04], buprenorphine [N02AE01], pethidine [N02AB02], and pentazocine [N02AD01].

Over the 13 years, the relative proportions of the nine pain-related conditions analyzed remained stable for the main weak opioid subtypes (tramadol and codeine) as well as for tapentadol and the category of other opioids (dihydrocodeine, hydromorphone, dextropropoxyphene, buprenorphine, pethidine and pentazocine) (Figure 3). Back and neck pain represented more than 50% of the indications of weak opioids (tramadol and codeine) and of tapentadol which is an opioid classified as strong (Anekar and Cascella, 2020). Strong opioids such as fentanyl, morphine and oxycodone were more frequently indicated for cancer, however, there is an increasing trend of use of these strong opioids to treat non-cancer medical conditions; For example, the non-cancer indications for morphine merely comprised 29% of any pain-related medical conditions in 2007, but this figure gradually increased to 60% in 2019.

## Discussion

### Main Findings

Based on a representative cohort of adults covering over 80% of the population in Catalonia, we observed that the use of opioids was relatively stable from 2007 to 2013, but increased sharply after that until 2019. These increases dominated particularly in older adults and were even more pronounced when accounting for opioid strengths. Regarding the opioid indications, this report shows increasing use of strong opioids (such as morphine, fentanyl and oxycodone) to treat non-cancer medical conditions.

### Possible Factors Contributing to the Rise/Explanations

Several factors might underline the recent rising trends of opioid use in Catalonia amid the global calling for early and active treatment of pains.

First, the ageing of the population with the consequent increase in chronic painful medical conditions, such as osteoarthritis, could have contributed to the overall increase in the opioid dispensations observed as effective medications for pain management in the elderly is highly needed. Also, poor modern lifestyle habits and the expanded occupational risk factors could have also contributed to the high proportion of back and neck pains observed in our study, potentially leading to increased use of both weak and strong opioids (Shiri et al., 2019). Safety concerns regarding the potential cardiovascular risks for the use of non-steroidal anti-inflammatory drugs (NSAIDs) might have facilitated clinicians to prescribe opioids as an alternative therapy in this population (Kalkman et al., 2019). Two meta-analyses published in 2011 and 2013 found that almost all NSAIDs were associated with a higher risk of cardiovascular events such as myocardial infarction, stroke, or cardiovascular death (Trelle et al., 2011). Even though uncertainty remains, it is still recommended that cardiovascular risk be considered when prescribing NSAIDs (Schmidt et al., 2016; Schjerning et al., 2020).

Second, the observed growth of the opioid dispensations has been even more pronounced since 2013, which coincides with changes in the regulatory framework to facilitate opioid prescribing (integrating these opioids to the electronic prescription and extending its renewal from quarterly to annually) (Agencia Estatal Boletín Oficial del Estado, 2020). Although more research is needed to quantify the impact of this reform on the clinical practice of opioid prescription in Spain, changes in policy regulations have been previously identified in other countries as responsible for the increase in opioid prescriptions (Musazzi et al., 2018).

In this study, we found that tramadol was the only type of opioid that experienced a consistent increase during the entire period from 2007 to 2019, which is in line with previous national reports that showed a 10-fold increase in 5 years from 1993 to 1998 (del Pozo et al., 1999). Since first licensed in Spain, tramadol had been marketed as an “atypical” opioid with fewer side effects and addiction potential compared to other opioids, which could explain the increase in dispensations observed. Tramadol and codeine are both classified as “weak” opioids and could be prescribed for similar indications, although our recent research showed a noticeable difference in their safety profile (Xie et al., 2021). In this study, we found that the number of users and dispensations of these two opioids varied substantially, with a stable decrease of codeine use after 2013. This difference can be again explained by changes in governmental policies in 2012 (Agencia Estatal Boletín Oficial del Estado, 2020) where many products containing codeine were no longer funded by the Spanish social security, leading patients to seek another similar pain killer such as tramadol.

### Opioid Pandemic

The increased rate of opioid dispensations in Catalonia is similar to reported in other regions of Spain (Herrera-Gómez et al., 2019; Hurtado et al., 2020). A recent cross-sectional study, carried out in a mid-eastern region of Spain, analyzed the opioid prescription and MME from 2010 to 2018 (Hurtado et al., 2020). During this period, they found that the MME of tramadol increased 2 folds, fentanyl more than tripled, and tapentadol was the second in terms of MME in 2018. Our results go in the same line, with a much more pronounced increase in MME for tramadol (increasing more than 7 folds from 2007 to 2019), fentanyl (increasing more than 10 folds from 2007 to 2019), and tapentadol (increasing more than 160 folds from very low levels after its commercialization in 2011).

As in this study, other European reports have also observed an overall increasing trend in opioid use in the last decade (Hider-Mlynarz et al., 2018; Bosetti et al., 2019; Kalkman et al., 2019; Pierce et al., 2021). Methodological differences of these studies render comparison difficult, and their results also vary on the type of opioid that drives the increase. For the Netherlands (Kalkman et al., 2019), this increase is at the expense of oxycodone (with a 4-fold increase from 2007 to 2017). In the United Kingdom, buprenorphine and fentanyl are the ones responsible for the increase between 2008 and 2018 (Pierce et al., 2021). Fentanyl is the opioid most consumed in western, norther EU countries and Spain between 2014 and 2016 (Bosetti et al., 2019). Results on the use of tramadol and codeine are also uneven; while tramadol use declined in the Netherlands especially from 2013 to 2017 (Kalkman et al., 2019), in France tramadol and codeine increased a 62 and 42% from 2006 to 2015 (Hider-Mlynarz et al., 2018) respectively. Our study accounted for all the opioids commercialized in Spain including tramadol and codeine which are commonly used as the second step in the WHO analgesic ladder. Results show that tramadol was responsible for the increase both in the number of dispensations and total MME.

Although the opioids use in Catalonia is still far from what is reported in the US (Guy et al., 2019), the increasing trend of the opioid dispensations and MME reported in this study, which is similar to what has been published in other regions of Spain, foresees a future health care problem in this country. The opioid epidemic is a multifaceted crisis that requires the coordinated action of both governmental regulations and clinicians. The sharper increase of the opioid dispensations reflected in our data from 2013 onwards, which coincides with a loosening of the opioid prescription’s regulation suggests that prevention strategies should start here. More stringent opioid regulations have been already implemented in the US, and recent reports attribute it to the decreasing trend in the opioid prescriptions seen in recent years (Schieber et al., 2020).

### Strengths and Limitations

The main strength of this study is the use of a representative cohort of more than 4.5 million patients in Catalonia and the use of real-world data of primary health care centres with detailed drug dispensation information, including ATC code, dose, unit, and duration.

Several limitations warrant consideration in this study. First, our data provide no information on opioid-related complications, overdose, illicit opioid use, or mortality. Evidence on both opioid dispensations and side effects is crucial to weigh opioid risks and benefits to public health. While an increase in opioid dispensations may reflect more significant attention to effective pain management or patients’ improved expectations of pain relief, the increase in total MME might reflect the burden of opioid exposure in our population.

Second, the retrospective review of pharmacy dispensing data has limitations; we were unable to assess direct drug compliance and therefore all drug dispensed was assumed to be used. However, we expect that the non-compliance rate is stable over the study period and would have little impact on our estimate of trends. Furthermore, compared to most prior population-level-based studies that used prescribing databases to surrogate opioid consumption, our study using dispensed prescriptions is unlikely to be subject to overestimation of opioid use due to unfilled prescriptions.

Third, the opioid dispensations were not automatically linked to a specific medical condition and although an operational definition was defined a priori to assess the indications, the risk of misclassification still exists. Such information is of great value for distinguishing opioids used for acute pain, cancer pains, or chronic non-cancer pain and understanding the driving force behind the increase.

Fourth, we only accounted for dispensations prescribed in the community pharmacies and were not able to cover those extended at the hospital level, with the consequent risk of underestimating the real opioid consumption. However, these would mostly affect acute treatments as chronic treatments are regulated by primary care clinicians and therefore would be captured at some point in our database.

In conclusion, our findings show a considerable increase in opioid dispensations and MME among the elderly population over 2007–2019 and a shift in the opioid indications towards using strong potent opioids to treat non-cancer pain. Authorities should implement more rigorous opioid stewardship, particularly among older people, to prevent further escalation in Catalonia.

## What Is Already Known on This Topic

1) Opioid use, addiction, related morbidity, and mortality are still rising in the United States, despite the comprehensive strategies implemented to address the opioid epidemic.
2) The increasing use of opioids has been mainly reported in Northern and Central Europe, but there is a scarcity of data regarding the Southern countries.
3) Previous studies have noted that accounting for opioid strength is crucial for monitoring opioid consumption but is often overlooked.

## What This Study Adds

1) This study found a 12, 105, and 339% increase in opioid users, dispensations and morphine milligram equivalent doses from 2007 to 2019 in Catalonia (Spain).
2) The substantial increase of opioids use among older adults and its progressively expanded indications for non-cancer pains were the driving factors for the observed escalation trend.
3) The results suggest an opioid prescription transition from weak to strong and from intermittent to chronic, which calls for more rigorous stewardship of opioids prescribed for non-cancer pains, particularly in the older population.

## Data Availability

The datasets presented in this article are not readily available because the data was obtained from the Information System for Research in Primary Care - SIDIAP. Requests to access the datasets should be directed to: https://www.sidiap.org/index.php/solicituds.
