# Supplementary material for: Trends of Dispensed Opioids in Catalonia, Spain, 2007–19: A Population-Based Cohort Study of Over 5 Million Individuals
Source: Front Pharmacol. 2022 Jun 8;13:912361. doi: 10.3389/fphar.2022.912361 (PMC9213744; doi:10.3389/fphar.2022.912361)
Supplement: Supplementary file 1 [file Table1.pdf]

Table A1: ICD-10 codes for the identification of nine pain-related conditions

Osteoarthritis	M18.9, M17.9, M47.9, M18.10, M15.9, M47.899, M19.90, M19.049, M15.0, M19.039, M16.9, M18.0, M15.1, M17.10, M15.2, M15.4, M17.0, M16.0, M16.10, M15.8, M15.3, M17.30, M16.30, M16.50, M16.6, M18.50, M16.7, M17.4, M17.2, M16.2, M18.2, M16.4, M18.30, M19.071, M17.5, M19.019, M19.271, M19.079, M19.011, M19.072, M19.041, M19.172, M19.111, M19.029, M19.012, M18.4, M19.119, M19.219, M19.272, M19.279, M19.171, M19.042, M19.112, M19.141, M19.149, M19.031, M19.021, M19.179, M19.0, M19.032, M19.242, M19.2, M19.241, M19.022
Fibromyalgia	M79.7
Back pain	M54.5, M54.9, M54.89, M51.26, M54.40, M54.2, M43.10, M51.9, M54.30, M54.10, M43.00, M54.6, M53.1, M53.3, M43.8X9, M43.9, M51.24, M43.20, M53.9, M51.34, M51.17, M51.86, M51.16, M43.3, M53.0, M51.06, M51.47, M51.05, M51.25, M53.80, M51.36, M43.5X9, M51.46, M51.35, M51.84, M51, M51.37, M51.2, M43.4, M51.27, M51.15, M51.45, M51.87, M51.85, M51.1, M51.0
Neck/shoulder pain	M43.6, M54.2, M25.50, M50*, M53.0, M53.1,
Cancer	C00*-D49*
Bone fractures	S62.90XA, T14.8, T07, S92.309A, S52.90XA, S22.009A, S22.39XA, S32.10XA, S72.90XA, S62.309A, S42.009A, S62.109A, S42.309A, S82.209A, S82.009A, S72.009A, S82.409A, S92.009A, S42.409A, S32.009A, S82.90XA, S22.20XA, M80.80XA, S82.899A, S62.009A, S32.2XXA, S32.9XXA, S42.209A, S82.66XA S42.109A, S82.56XA, S62.209A, S82.109A, S72.109A, S32.309A, S92.109A, S22.49XA, S82.309A, S72.26XA, S32.409A, S32.509A S72.409A, S72.309A, S22.5XXA, S72.8X9A
Cough	R05
Falls	W19.XXXA, W00.0XXA, W01.0XXA, W06.XXXA, W10.9XXA, W08.XXXA, W10.1, W03.XXXA, W00.9XXA, W18.39XA, W01.0, W16.42XA, W12.XXXA, W07.XXXA, W15.XXXA, W05.0XXA, V00.891A, W14.XXXA, W11.XXXA, W17.89XA, W18.30, W13.9XXA, W09.8XXA, W18.01XS, W04.XXXA, W13.3XXS, W13.3XXD, W13.3XXA, W10.8, W10.1XXS, W13.3, W01.198D, W10.8XXS, W18.31XS, W18.30XA, W18.09, W17.81, W06.XXXD, W18.30XD, W13.2XXA, W01.198A, W01.10XS, W18.30XS, W18.39, W00.0XXS, W10.8XXD, W06, W10.8XXA, W01.190A
Major surgeries (Joint replacement, limb amputation)	Z96.6, Y83.5

**All subcodes included*
