# Supplementary material for: Trends of Dispensed Opioids in Catalonia, Spain, 2007–19: A Population-Based Cohort Study of Over 5 Million Individuals
Source: Front Pharmacol. 2022 Jun 8;13:912361. doi: 10.3389/fphar.2022.912361 (PMC9213744; doi:10.3389/fphar.2022.912361)
Supplement: Supplementary file 2 [file Table2.pdf]

Table A2: Basic demographic information of entire registered residents in SIDIAP by year.

Study year	Registered residents	Age	Sex		Living area			Socioeconomic status					
	Number	Mean (SD)	Female (%)	Male (%)	Urban (%)	Rural (%)	Missingness (%)	U1 (%)	U2 (%)	U3 (%)	U4 (%)	U5 (%)	Missingness (%)
2007	4,656,197	47.3 (18.4)	2,376,117 (51.0)	2,280,080 (49.0)	3,765,841 (80.9)	876,934 (18.8)	13,422 (0.3)	662,894 (14.2)	645,092 (13.9)	648,310 (13.9)	641,566 (13.8)	617,919 (13.3)	1,440,416 (30.9)
2008	4,791,568	47.4 (18.4)	2,440,103 (50.9)	2,351,465 (49.1)	3,884,031 (81.1)	893,012 (18.6)	14,525 (0.3)	681,906 (14.2)	662,310 (13.8)	665,906 (13.9)	661,993 (13.8)	643,374 (13.4)	1,476,079 (30.8)
2009	4,903,562	47.6 (18.4)	2,491,639 (50.8)	2,411,923 (49.2)	3,985,097 (81.3)	905,678 (18.5)	12,787 (0.3)	698,032 (14.2)	676,642 (13.8)	681,163 (13.9)	679,506 (13.9)	666,174 (13.6)	1,502,045 (30.6)
2010	4,963,931	47.8 (18.4)	2,520,563 (50.8)	2,443,368 (49.2)	4,040,398 (81.4)	911,924 (18.4)	11,609 (0.2)	708,803 (14.3)	686,299 (13.8)	689,124 (13.9)	689,437 (13.9)	678,314 (13.7)	1,511,954 (30.5)
2011	4,966,548	48.2 (18.5)	2,522,892 (50.8)	2,443,656 (49.2)	4,044,544 (81.4)	910,754 (18.3)	11,250 (0.2)	709,464 (14.3)	688,071 (13.9)	691,783 (13.9)	694,016 (14.0)	682,616 (13.7)	1,500,598 (30.2)
2012	4,966,393	48.6 (18.5)	2,523,389 (50.8)	2,443,004 (49.2)	4,046,763 (81.5)	908,734 (18.3)	10,896 (0.2)	714,658 (14.4)	691,936 (13.9)	693,974 (14.0)	694,908 (14.0)	685,222 (13.8)	1,485,695 (29.9)
2013	4,875,535	49.0 (18.5)	2,481,589 (50.9)	2,393,946 (49.1)	3,970,599 (81.4)	896,281 (18.4)	8,655 (0.2)	706,661 (14.5)	689,637 (14.1)	691,132 (14.2)	690,674 (14.2)	679,395 (13.9)	1,418,036 (29.1)
2014	4,855,506	49.3 (18.5)	2,473,821 (50.9)	2,381,685 (49.1)	3,953,824 (81.4)	893,162 (18.4)	8,520 (0.2)	710,235 (14.6)	692,605 (14.3)	691,730 (14.2)	687,668 (14.2)	677,613 (14.0)	1,395,655 (28.7)
2015	4,790,736	49.6 (18.5)	2,443,679 (51.0)	2,347,057 (49.0)	3,902,606 (81.5)	879,670 (18.4)	8,460 (0.2)	704,882 (14.7)	690,489 (14.4)	688,442 (14.4)	682,102 (14.2)	669,193 (14.0)	1,355,628 (28.3)
2016	4,772,900	49.9 (18.5)	2,437,454 (51.1)	2,335,446 (48.9)	3,888,962 (81.5)	875,391 (18.3)	8,547 (0.2)	709,429 (14.9)	694,429 (14.5)	690,761 (14.5)	683,864 (14.3)	670,648 (14.1)	1,323,769 (27.7)
2017	4,775,950	50.1 (18.6)	2,442,100 (51.1)	2,333,850 (48.9)	3,893,455 (81.5)	873,785 (18.3)	8,710 (0.2)	715,084 (15.0)	700,415 (14.7)	695,413 (14.6)	688,785 (14.4)	676,535 (14.2)	1,299,718 (27.2)
2018	4,786,105	50.3 (18.6)	2,449,613 (51.2)	2,336,492 (48.8)	3,906,884 (81.6)	871,314 (18.2)	7,907 (0.2)	723,349 (15.1)	708,867 (14.8)	702,914 (14.7)	696,312 (14.5)	686,786 (14.3)	1,267,877 (26.5)
2019	4,798,114	50.4 (18.6)	2,456,520 (51.2)	2,341,594 (48.8)	3,922,138 (81.7)	868,051 (18.1)	7,925 (0.2)	731,733 (15.3)	717,815 (15.0)	711406 (14.8)	705876 (14.7)	699,897 (14.6)	1,231,387 (25.7)

SD: Standard Deviation; Socioeconomic status: U1=Least deprived, U5=Most deprived
