# Supplementary material for: Trends of Dispensed Opioids in Catalonia, Spain, 2007–19: A Population-Based Cohort Study of Over 5 Million Individuals
Source: Front Pharmacol. 2022 Jun 8;13:912361. doi: 10.3389/fphar.2022.912361 (PMC9213744; doi:10.3389/fphar.2022.912361)
Supplement: Supplementary file 3 [file Table3.pdf]

Table A3: The percentage changes of opioid use between 2007 and 2019 among subgroups by age, sex, living area and socioeconomic status

	**Number of users per 1,000 population**			**Number of dispensations per 1,000 population**			**Oral MME per 1,000 population**		
	2007	2019	PC (%)	2007	2019	PC (%)	2007	2019	PC (%)
**Age**									
[18,25)	36.41	10.53	-71	54.52	18.14	-67	7,677	1,715	-78
[25,30)	31.86	15.36	-52	47.71	27.12	-43	7,020	3,463	-51
[30,35)	32.29	18.67	-42	48.80	35.61	-27	7,622	7,289	-4
[35,40)	34.40	24.70	-28	52.40	51.79	-1	8,633	14,842	72
[40,45)	35.85	31.29	-13	56.12	72.72	30	9,717	25,609	164
[45,50)	38.50	37.62	-2	63.46	95.83	51	12,135	37,985	213
[50,55)	42.09	47.07	12	71.94	133.81	86	13,790	58,490	324
[55,60)	46.77	55.43	19	82.07	172.66	110	15,358	75,822	394
[60,65)	50.18	62.77	25	92.15	203.39	121	18,201	85,175	368
[65,70)	50.75	67.16	32	100.17	220.48	120	20,724	88,325	326
[70,75)	48.04	73.18	52	100.44	259.59	158	21,550	104,054	383
[75,80)	41.34	75.83	83	88.90	293.16	230	19,083	120,296	530
>= 80	27.46	71.99	162	63.16	331.01	424	14,558	135,364	830
**Sex**									
Female	42.04	50.28	20	74.19	166.45	124	13,977	66,744	378
Male	34.49	35.33	2	57.41	102.78	79	10,291	39,448	283
**Living area**									
Rural	42.28	44.55	5	73.45	142.70	94	13,914	58,170	318
Urban	37.48	42.67	14	64.32	133.85	108	11,783	52,400	345
Missingness	24.51	26.75	9	40.60	87.82	116	7,493	39,424	426
**Socioeconomic status**									
U1	29.34	30.03	2	50.62	96.05	90	9,190	37,674	310
U2	38.24	39.90	4	65.12	126.10	94	11,793	48,418	311
U3	41.52	44.86	8	71.17	140.43	97	12,760	53,726	321
U4	42.83	49.89	16	72.45	152.67	111	13,108	59,032	350
U5	42.01	51.15	22	70.73	157.32	122	12,623	62,818	398
Missingness	37.54	42.79	14	66.16	138.85	110	12,840	56,968	344

PC: Percent Change; MME: Morphine milligram equivalents; Socioeconomic status: U1=least deprived, U5=most deprived
